# Anti-Eryptotic Activity of Food-Derived Phytochemicals and Natural Compounds

**DOI:** 10.3390/ijms23063019

**Published:** 2022-03-11

**Authors:** Ignazio Restivo, Alessandro Attanzio, Luisa Tesoriere, Mario Allegra, Guadalupe Garcia-Llatas, Antonio Cilla

**Affiliations:** 1 Department of Biological, Chemical and Pharmaceutical Sciences and Technologies, University of Palermo, Via Archirafi 28, 90123 Palermo, Italy; ignazio.restivo@unipa.it (I.R.); luisa.tesoriere@unipa.it (L.T.); mario.allegra@unipa.it (M.A.); 2 Nutrition and Food Science Area, Faculty of Pharmacy, University of Valencia, Avda, Vicente Andrés Estellés s/n, 46100 Burjassot, Spain; guadalupe.garcia@uv.es (G.G.-L.); antonio.cilla@uv.es (A.C.)

**Keywords:** eryptosis, phytochemicals, food-derived compound, red blood cells, phenolic compounds, alkaloids, oxidative stress

## Abstract

Human red blood cells (RBCs), senescent or damaged due to particular stress, can be removed by programmed suicidal death, a process called eryptosis. There are various molecular mechanisms underlying eryptosis. The most frequent is the increase in the cytoplasmic concentration of Ca^2+^ ions, later exposure of erythrocytes to oxidative stress, hyperosmotic shock, ceramide formation, stimulation of caspases, and energy depletion. Phosphatidylserine (PS) exposed by eryptotic RBCs due to interaction with endothelial CXC-Motiv-Chemokin-16/Scavenger-receptor, causes the RBCs to adhere to vascular wall with consequent damage to the microcirculation. Eryptosis can be triggered by various xenobiotics and endogenous molecules, such as high cholesterol levels. The possible diseases associated with eryptosis are various, including anemia, chronic kidney disease, liver failure, diabetes, hypertension, heart failure, thrombosis, obesity, metabolic syndrome, arthritis, and lupus. This review addresses and collates the existing ex vivo and animal studies on the inhibition of eryptosis by food-derived phytochemicals and natural compounds including phenolic compounds (PC), alkaloids, and other substances that could be a therapeutic and/or co-adjuvant option in eryptotic-driven disorders, especially if they are introduced through the diet.

## 1. Eryptosis

The average life span of circulating mature human erythrocytes is about 100–120 days. The elimination of senescent RBCs is mediated by the activation of the complement, by the Band-3 protein [1], and the hemolytic process is carried out by macrophages in the spleen. Human RBCs damaged under particular stress can be removed before senescence by programmed suicidal death, a process called eryptosis [2]. Until recently, it was thought that because they lack a nucleus and mitochondria, RBCs were unable to undergo programmed cell death. This hypothesis has been denied by much experimental evidences that has demonstrated the existence of the process known as eryptosis, which regulates the elimination of damaged or senescent RBCs [3]. Eryptosis is, therefore, a physiological process, mostly similar to the apoptosis of nucleated cells. From a physiological point of view, eryptosis is a very important process as it prevents intravasal hemolysis of RBCs, allowing their elimination without, however, leading to rupture of the membranes and consequent inflammation due to the release of intracellular contents [4]. Eryptosis on the one hand, and hematopoietic processes on the other, play a fundamental role in maintaining an adequate number of circulating erythrocytes. Any de-regulation of these processes can alter the number of circulating erythrocytes, compromising the oxygenation capacity of the tissues. In healthy subjects, the hematopoietic processes mediated by the bone marrow and the hemocateretic processes mediated by macrophages that lead to the elimination of senescent or damaged RBCs, are in constant equilibrium. This balance is finely regulated by erythropoietin [5]. Therefore, the most important signs of eryptosis are cell shrinkage, membrane blebbing, and exposure of phosphatidylserine to the outer membrane sheet, characteristics that are completely stackable to the apoptotic process.

### 1.1. Mechanisms of the Eryptotic Machinery

**Figure 1:**
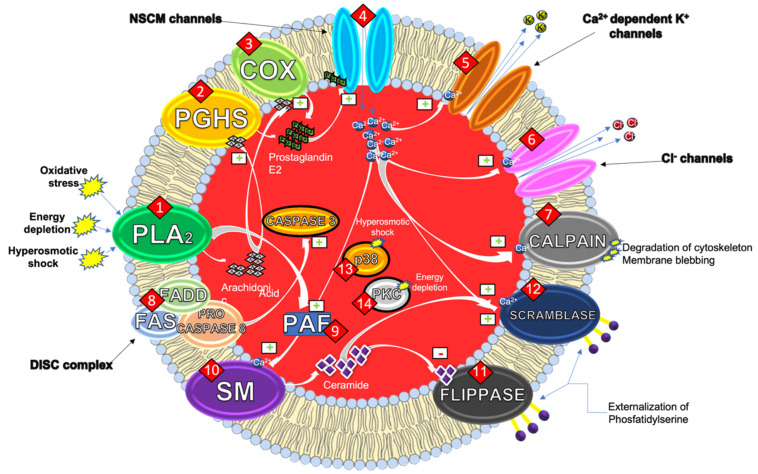
Biomolecular pathways that regulate the eryptosis machinery. The green plusses inside the rectangles indicate activation, the red minuses inside the rectangles indicate inhibition. The bold numbers inside the round brackets inserted in the text refer to the numbering of the detracted pathway in the figure. Modified version of figure present in [6].

There are several molecular mechanisms underlying eryptosis (Figure 1). One of the most relevant is related to the increase of endocellular calcium concentration.

Under physiological conditions, Ca^2+^ homeostasis is regulated through the complementary actions of both Ca^2+^ pumps, that act through ATP-dependent extrusion mechanisms, and Ca^2+^ channels, that allow extracellular Ca^2+^ to enters [6]. 

Pro-eryptotic stimuli activate phospholipase A2 (PLA2) that releases arachidonic acid (AA) (**1**). This latter is, then, metabolized by prostaglandin endoperoxide synthase (PGHS) (**2**), or cyclooxygenase (COX) (**3**), to prostaglandin E2 (PGE2). Ca^2+^ ions enter the cytosol of the erythrocyte through non-selective cationic channels, stimulated by PGE2 (**4**) [7]. 

The increase of cytosolic levels of Ca^2+^ ions then activates the calcium-dependent potassium (K^+^) channels (**5**) (also called Gardos Channels). The activation of these channels induces the release of K^+^ and Cl^−^ ions in the extracellular space (**6**) eventually leading to membrane hyperpolarization. The loss of these ions causes an osmotic flow that leads to the leakage of water and the consequent reduction of cell volume (shrinkage), typical of erythrocytes [8]. 

The increase of cytosolic Ca^2+^ levels also stimulates calpain (**7**), a cysteine endopeptidase, responsible for both degradation of RBC cytoskeleton and membrane blebbing that increases erythrocyte adhesiveness [6]. If caspases are the essential mediators of apoptosis in nucleated cells, µ-calpain is the protease responsible for the cleavage of various membrane proteins in erythrocytes producing significant morphological alterations of the cell [9]. 

Although in 2001 it was shown that erythrocyte caspases were functionally active ex vivo [10], the role of caspase 8 and caspase 3 in the eryptotic process was elucidated a few years later in a study in which caspases were shown to have a functional role in mature RBCs. As a result of the redox imbalance to which mature erythrocytes are subject, some phenomena involving caspases have been highlighted. One of the most important concerns the translocation of Fas receptor (FAS) to the membrane rafts with the formation of the death-inducing signaling complex (DISC) given by the trimerization of FAS, Fas associated via death domain (FADD) and procaspase 8 ends with the activation of caspase 3 (**8**) which together with the activity of calpain has effects on the cytoskeleton. These events are independent from the activation of calpain and stimulated by oxidizing species such as tert-butyl hydroperoxide or phenylhydrazine [11]. 

Furthermore, with the loss of Cl^−^, there is still a PGE2 discharge that further increases the intracellular Ca^2+^ levels. While PLA2 activation leads to AA release, on the other hand, it can also stimulate platelets activating factor (PAF) formation (**9**). PAF is a powerful scramblase stimulator and concurs to its activation together with PGE2. 

Together with the entrance of Ca^2+^, PAF also stimulates the activity of membrane sphingomyelinase (SM) (**10**). SMs are ubiquitous enzymes catalyzing the hydrolysis of cell membrane sphingomyelin and producing ceramide. This latter acts as a key, second intracellular messenger, both in cell differentiation and apoptosis. The increase of membrane ceramide and Ca^2+^ levels, in fact, determines the inhibition of flippases (**11**) and the activation of scramblases (**12**). These enzymes, unlike the scramblases, are selective transmembrane proteins: they remove certain phospholipids from the outer sheet of the membrane and flip them into the leaflet exposed to the cytosol. As a result of the activation of scramblases and the simultaneous inhibition of flippases, PS remains outsourced. This mechanism could play a key role in the characteristic hyper-adhesiveness of eryptotic erythrocytes, that eventually leads to the impairments of the microcirculation [12,13].

Other studies highlighted another mechanism involved in energy depletion-induced eryptosis, that reduces the activity of the calcium-ATPase with a consequent increase of endocellular Ca^2+^ concentration that triggers all the above-mentioned mechanisms. The lack of energy also reduces the enzymatic activity of glutathione reductase, preventing the regeneration of reduced glutathione (GSH) and depleting the antioxidant defenses of erythrocytes that become more susceptible to oxidative stress. Under physiological energetical conditions, PS is maintained in the inner leaflet of the membrane by flippases, whose activity is ATP-dependent. Consequently, a decrease in the activity of this transporters results in a greater amount of outsourced PS. In addition, other studies have shown an involvement of the protein p38 kinase (**13**) in the mechanism that triggers eryptosis following hyperosmotic shock [14]. Finally, energy depletion can lead to the activation of protein kinase C (PKC) (**14**), a serine/threonine kinase that stimulates eryptosis, by phosphorylating membrane proteins [15,16]. 

All the above-mentioned mechanisms seem to lead to the maintenance of PS on the outer leaflet of the membrane. The loss of membrane asymmetry and the PS externalization are key events in the stimulation of the phagocytic activity of macrophages that ensures the removal of erythrocytes from the bloodstream [17].

### 1.2. Pathophysiological Implications

**Figure 2:**
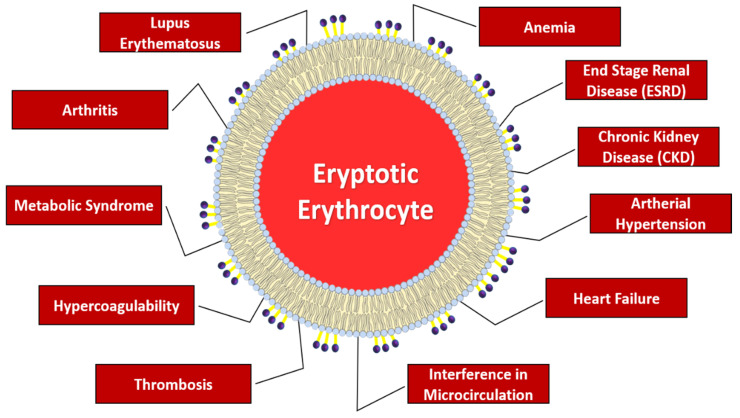
Main diseases related to an excess of eryptosis.

Several pathologies with relevant clinical significance are linked to excessive eryptosis, as summarized in Figure 2, mainly involving anemia, deranged microcirculation, and/or increased prothrombotic risk [6,18].

Anemia is one of the most frequent pathologies affecting RBCs, characterized, for the most part, by a decrease in levels of hemoglobin which leads to an insufficient supply of oxygen to the tissues. As mentioned above, as long as the eryptotic processes come compensated by adequate erythropoiesis, the number of erythrocytes circulating in the blood remains unaffected. In conditions where erythropoiesis is no longer able to counterbalance the accelerated loss of erythrocytes due to eryptosis, clinically evident anemia could result [2,4]. 

A study has highlighted the central role of eryptosis in anemia found in patients with terminal stage renal disease (end stage renal disease, ESRD). In particular, the erythropoiesis is compromised by the renal production and release of erythropoietin (EPO) [19] and from iron deficiency [20]. Trials suggest, however, that anemia in ESRD largely results induced by an accelerated clearance of circulating erythrocytes, due to stimulation of eryptosis [21]. Thus, the condition of anemia does not normalize despite the compensation of the EPO deficiency, and, therefore, the persistence of anemia is due to a greater extent to an increase of eryptosis [22]. Even in chronic kidney disease (CKD), that ultimately causes kidney failure, there is a condition of anemia. The cause of anemia was initially associated with an iron deficiency [23]. More recent evidence, however, suggests that the cause of the anemia in these patients is eryptosis which reduces the average life of circulating erythrocytes. 

Even liver failure and fibrosis are associated with anemia which can derive from various conditions, such as bleeding, malignant tumors, viral infections, chronic inflammation, and deficiency of essential nutrients like vitamin B12 and folate [24,25]. It has recently been shown that the resulting anemia from liver failure is also attributable to eryptosis, which is induced by high bilirubin levels. Indeed, the high levels of conjugated bilirubin in the blood can increase the cytosolic activity of Ca^2+^ and the formation of ceramide [26]. 

The average lifespan of RBCs is also shortened in diabetes [27]. Anemia is prevalent in a large number of diabetic subjects and cannot be attributed to a deficiency in erythropoiesis, since the number of reticulocytes is increased [28,29]. New studies have revealed that anemia related to diabetes can derive, at least in part, from an increase in eryptosis [30,31]. The erythrocytes of diabetic patients show an increase in the activity of superoxide dismutase and reactive oxygen species (ROS) production. The mechanism of diabetes-induced eryptosis has been identified in glycoxidation, which involves the glycation of biomolecules, dependent on oxidative stress. In fact, a condition of lasting hyperglycemia accelerates the glycation of the free amino groups of the membrane proteins in RBCs. This process leads to the formation and accumulation of advanced glycation end products (AGEs), substances with a pro-oxidant nature that induce morphological alterations of the RBCs cytoskeleton culminating in the increase in PS levels on the outer membrane [32,33]. 

A recent study has also highlighted the involvement of eryptosis in arterial hypertension. This is characterized by high arterial pressure, both systolic and diastolic. Usually, this pathology is associated with dyslipidemia, which consists of an alteration of the concentration of triglycerides or cholesterol in the blood. Both ailments can contribute to oxidative stress which, as we have already said, can induce an increase in eryptosis. The study involved four different groups: normotensive with and without dyslipidemia, and hypertensive with and without dyslipidemia. Hypertensive patients had higher eryptosis levels, associated with an increase in Ca^2+^ levels and oxidative stress; thus, suggesting that eryptosis participates in the pathophysiological mechanisms of hypertension [34]. 

Anemia is also a common condition in heart failure. According to a study, patients suffering from heart failure show an increased percentage of erythrocytes with externalization of PS, ROS levels and a reduced cell volume. Increased oxidative stress is one key feature of heart failure that can promote hemolysis and eryptosis [35]. 

In addition, eryptosis can interfere with microcirculation, as the erythrocytes that expose PS have a greater ability to adhere to the vascular wall [36]. The eryptotic erythrocytes, due to their procoagulant phenotype, can contribute to the onset of thrombosis.

**Figure 3:**
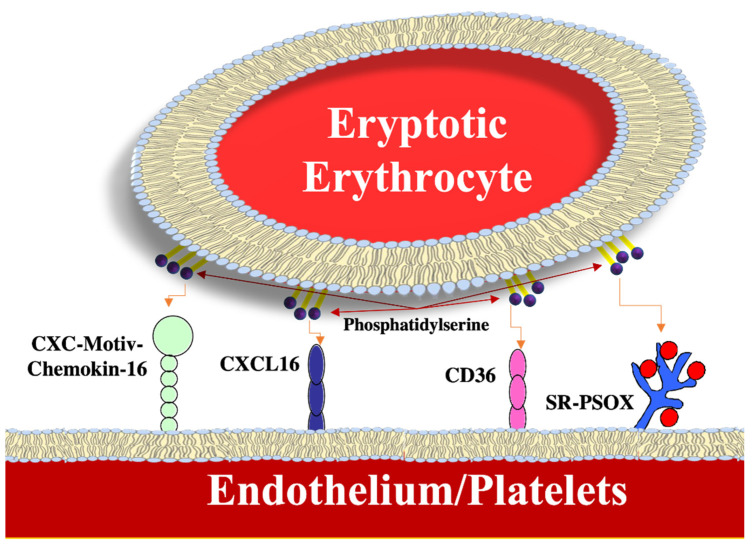
Exemplification of the adhesion of RBC to blood vessel or platelets.

Adherence to endothelial cells and platelets by erythrocytes that expose PS on their membranes is due to the ability to interact with certain surface receptors; for example, as shown in Figure 3, with the transmembrane ligand CXC chemokine CXCL16, also known as SR-PSOX. The latter acts as scavenger receptor with a high affinity for PS and with oxidized low-density lipoproteins. Platelets also express CXCL16 which stimulates their activation and translates vascular inflammation into thrombo-occlusive events [37]. Moreover, platelets express CD36, a receptor that was demonstrated to be able to interact with PS. The interaction of erythrocytes with platelets can promote thrombus formation in conditions of hypercoagulability associated with various clinical disorders, such as liver insufficiency and chronic kidney disease. In a model of thrombosis triggered by ferric chloride, erythrocytes have been shown to recruit platelets in the site of the injury, thus supporting the idea that they contribute actively to the pathophysiology of thrombosis [38]. The outsourcing of the PS on the cell membrane of erythrocytes serves as a platform for the assembly of prothrombinase (factor X) that stimulates the formation and the coagulation of thrombin, thus mediating the procoagulant effects of eryptotic erythrocytes. PS exposure can be further stimulated by the coagulation factors I, V, and X, which cause a state of hypercoagulability [39]. Likewise, smoking may aggravate microcirculation since it has recently been reported that cigarette smokers have a higher level of circulating erythrocytes than non-smokers characterized by greater externalization of PS, a decrease in intracellular stores of GSH and an increase in C reactive protein (CRP) [40].

Obesity is also linked to an increased risk of thrombosis. Although the mechanisms involved have not yet been elucidated, it is believed that a greater aggregability of erythrocytes and a reduced deformability of the same in obese patients cause hypercoagulability disturbances. In fact, in a recent study, it was shown that PS exposure in erythrocytes is significantly higher in patients with a higher body mass index than healthy subjects. Eryptosis participates in the hypercoagulability associated with obesity and to atherosclerosis, thus underlining the importance of the pathological link between erythrocytes and endothelial dysfunction and the activation of macrophages in obesity [41]. 

Many of the aforementioned factors can contribute to the development of the metabolic syndrome, a pathology that involves a series of metabolic factors that increase risks of cardiovascular diseases, diabetes, and associated diseases, such as dementia [6]. The development of the latter depends on the onset of systemic chronic inflammation. Indeed, the dysfunctional activation of the inflammatory response strongly compromises metabolic homeostasis of key tissues in energy use. A pro-inflammatory, pro-oxidant, and pro-thrombotic state has been observed in patients with metabolic syndrome. Accumulation of fat and obesity are major players in the development of chronic inflammation due to the ability of adipocytes to secrete pro-inflammatory mediators, such as cytokines or certain hormones like leptin, in response to hypertrophic signals. The persistence of the inflammatory state can induce endothelial dysfunction, which represents one of the early events which then leads to atherosclerosis. Clinical evidence suggests that eryptosis plays a key role in the development of all risk factors associated with metabolic syndrome, such as hyperglycemia, dyslipidemia, hypertension, and obesity, and clinical complications, such as diabetes and atherosclerosis [6]. This evidence is reinforced recently by an in vivo study that showed a higher level of eryptotic erythrocytes in hypercholesterolemic patients compared to normocholesterolemic [42]. In this sense, a recent ex vivo assay has suggested that saturated fatty acids (in particular lauric acid), well-known compounds that raise circulating cholesterol levels, induce eryptosis through a Ca^2+^-dependent externalization of PS, cell shrinkage and granularity, oxidative stress, accumulation of lipid peroxides, and stimulation of casein kinase 1α [43].

Epidemiological studies suggest that inflammation is also one of the major causes of anemia in the elderly and chronic pathologies. Anemia in inflammatory diseases is largely due to reduced iron homeostasis and suppression of erythropoiesis by pro-inflammatory cytokines. It has recently been shown that the inflammatory cytokines induce changes in the erythrocyte membrane. A study showed that anemia in arthritis patients is due to, at least in part, the increase in eryptosis. The latter is induced by an increased oxidative stress and increased cytosolic Ca^2+^ levels. It has been hypothesized that the increased adhesion of eryptotic erythrocytes to the endothelial vascular cells can contribute to the pathophysiology of vascular occlusion and ischemia in patients with arthritis [44]. 

Anemia also occurs in approximately 50% of patients with systemic lupus erythematosus (SLE), an autoimmune disease. Anemia in SLE has a multifactorial etiology and it can also be triggered by autoimmune destruction of erythrocytes and from immune-mediated hematopoietic insufficiency. Furthermore, in some patients with SLE, antibodies against erythropoietin were found, a fact by which they modify the normal production of erythrocytes. Anemia in SLE can also be caused by the reduced lifespan of erythrocytes since a significantly high percentage of circulating erythrocytes exhibit PS on their membranes. Furthermore, these patients show an increase of circulating reticulocytes, an increase in the cytosolic activity of Ca^2+^ and a high ROS production. Hence, eryptosis can contribute to pathophysiology anemia in SLE [45].

## 2. Natural Substances with Anti-Eryptotic Activity

Some natural or phytochemical products are effective ex vivo and in vivo on animals in contrasting eryptosis, that, as aforementioned, can be associated with different diseases [18]. Moreover, these compounds could be associated to anticancer drugs as the inhibition of eryptosis could limit the anemic state induced by chemotherapy [46]. 

The structural diversity of these substances made it possible to inhibit the eryptosis induced by various stimuli by acting on different biomolecular targets. Several studies focus on the evaluation, through different ex vivo models of RBCs and some animal models, of the major pro-eryptotic markers, such as PS externalization, increase of intracellular ROS and Ca^2+^, inhibition of cellular reserves of GSH, variation of cell’s volume, and caspase activation.

Compounds such as phenols, alkaloids, and other natural substances have an established positive effect in the treatment and prevention of oxidative stress and inflammation [47,48]. Therefore, deepening on the role they play in the fight against eryptosis could be an important step in the treatment and/or co-adjuvant strategies for eryptotic-related pathologies. 

### 2.1. Phenolic Compounds 

PCs are phytochemicals found in most plant tissues, including fruit and vegetables and, therefore, widely consumed especially in populations following the Mediterranean diet since it is rich in cereals, fruit and vegetable species, and olive oil, all foods rich in polyphenols [49]. PCs possess numerous bioactive properties and, although they are not nutritious, their dietary intake provides protective health effects like antioxidant effects that which help to inhibit the evolution of several serious diseases, such as cancer, Alzheimer’s, and diabetes [50]. Especially for Alzheimer’s, there are many studies that show the beneficial effects of PCs through their interaction with transition metals, inactivation of free radicals, inhibition of the inflammatory response, modulation of the activity of various enzymes and entering in the intracellular signaling pathways, and the expression of genes of interest [51].

Their anti-eryptotic effect has also been studied since 2009, and from the studies analyzed (see Table 1), several features common to many phenols that are able to inhibit eryptosis induced by particular stimuli emerge.

One of the most used pro-eryptotic stimuli is the oxidative stress induced by treating RBCs with 0.3 mM tert-butylhydroperoxide (tBOOH) for 30 min; to this stimulus, phenols such as PYR [56], RES [59], NAR [60], PHL [61], XAN [62], and THY [63], at physiological concentrations (0.1–100 µM), inhibit eryptosis always with the reduction of PS on the external side of the cell membrane, blunt of Ca^2+^, and often with the recovery of a normal cell volume evaluated cytofluorimetrically as Forward Scatter (FS). Other stimuli inducing oxidative stress in RBCs are 2- phenethyl isothiocyanate (PEITC), methyl glyoxal (MGO), oleoyl-L α-lysophosphatidic acid sodium salt (LPA), HgCl_2_, D-galactose, and cyadox. To counter these stimuli, PCs such as WGN against PEITC [52], ABE in countering the effect of MGO in an experimental in vivo model in zebrafish [53], RES to counteract the effects of cyadox in rabbits [58], FIS due to the high consumption of D-galactose in male wistar rats [57], and HT effective against LPA [54] and HgCl_2_ µM [55] can be effective. In addition, these compounds are also able to normalize the levels of ROS and GSH, as well as the activities of caspases 3 and 8 [58], activity of LDH [58], and the release of micro vesicles (MV) [55].

Another stimulus frequently used in the induction of eryptosis is the energy depletion given by the absence of glucose in the RINGER medium used for RBCs culture. The depletion of energy in addition to leading the RBCs to externalize the PS, often acts in the shrinkage of the cell volume and in the alteration of the levels of intracellular Ca^2+^. To counteract the pro-eryptotic effects of this stimulus WGN [52], PYR [56], RES [59], NAR [60], PHL [61], XAN [62], and THY [63] are particularly effective.

Against the cell shrinkage induced by a hyperosmotic environment caused by sucrose, the most effective PCs are RES [59], THY [63], WGN [52], and PYR [56]. The latter two polyphenols appear to protect RBCs also from eryptosis induced by Ca^2+^ imbalances given by the treatment with Ionomycin 1 µM for 1 h.

This anti-eryptotic effect is dependent of the type and structure of polyphenols since other studies have reported a pro-eryptotic action of, for instance, licochalcone, carnosic acid, apigenin, curcumin, and tannic acid [64,65]. Thus, more studies on structure-activity relationship on phenols and eryptosis are warranted.

### 2.2. Alkaloids Compounds 

Alkaloid compounds (ACs) occur primarily as a class of nitrogen-containing organic compounds in plants, fungi, and bacteria. They possess significant biological assets, often being one of the most important active ingredients in phytotherapy. The vast majority of alkaloids are present in higher plants such as dicotyledons. With advances in the separation of natural products and the continuous emergence of new technologies and methods, the development of alkaloid chemistry has expanded [66]. Alkaloids can be classified by source combined with chemical structures and find use especially as analgesics, cough suppressants, muscle relaxants, antimicrobials, and as precursors to semi synthetic drugs [67]. Table 2 shows the anti-eryptotic studies performed with ACs.

The first study concerning the anti-eryptotic activity of ACs dates to 2008. The study of Floride et al. [68] showed that CAF, in a range of concentrations between 50–500 µM, is able to inhibit the eryptosis induced by energy depletion and cell shrinkage with an inhibition of the externalization of PS and the restoring of normal values of FS and intracellular Ca^2+^. More recent studies instead showed that CHE inhibits eryptosis induced by custonolide with the restoring of a normal cell volume [69] and that IND at 1–5 µM (nutritional-relevant concentration) counteracts eryptosis induced by oxidative stress caused by mixture of oxysterols with the restoring of a normal oxidative balance and normal levels of Ca^2+^, FS and PGE2 [70].

### 2.3. Other Natural Compounds 

Nature is very rich in many other compounds that are able to inhibit the eryptosis induced by the most diverse stimuli. As shown in Table 3, acetylsalycilic acid (ASA) and tamarind seed extract (TSCEE) inhibit erytosis with the restoration of normal levels of intracellular Ca^2+^ [71,72]. Moreover, a mixture of plant sterols (MPS), L-carnitine (LCar), and salidroside (SAL) counteract eryptosis induced by oxidative stress caused by different inductors such as t-BOOH, uremic serum, and H_2_O_2_, respectively [73,74,75]. Vitamin C (VitC) with its ability to insert into the membrane and neutralize oxidative stress, manages to reduce the effects of energy depletion, cell shrinkage and oxidative stress [76,77] Finally, in rabbit’s RBCs, cinnamaldehyde (CIN) inhibited eryptosis induced by cyadox with the reduction of the activities of caspases 8 and 3 [58].

## 3. Conclusions

The role of food-derived phytochemicals and natural compounds in contrasting eryptosis ex vivo and in animals is, therefore, increasingly affirmed, although further structure–activity studies in vitro and in vivo depth studies in humans, are needed to corroborate these promising pre-clinical results. The main mechanisms of action to face the eryptosis machinery are the decrease in PS exposure, calcium influx, ROS overproduction, ceramide overproduction, and caspase activity, as well as the recovery of cell size and restoration of GSH levels. Therefore, inhibition of eryptosis with these bioactive compounds may be a novel therapeutic and/or co-adjuvant option to prevent the onset of all the disease caused by the higher grade of circulating erythrocytes. Thus, the inclusion of higher amounts of plant-derived foods in the regular diet may provide a cost-effective and preventive protective environment against eryptosis.

## Figures and Tables

**Table 1:** Mechanisms involved in the anti-eryptotic effect of polyphenolic compounds against different stimuli in different type of RBCs.

Phytochemical Compound	Concentration	Treatment Time	Inductor	References
**Wogonin (WGN)**	100 µM	24–48 h	Energy depletion—48 h Cell shrinkage—24 h Ionomycin 2 µM—24 h PEITC 134 mM—24 h	[52]
	
	
	
**Cellular mechanism** At 48 h with WGN, in presence of energy depletion, eryptosis inhibited with PS↓, Ca^2+^↓ and FS↑. At 24 h in presence of oxidative stress inducted by PEITC and hyperosmotic conditions, eryptosis inhibited with PS↓ and in presence of ionophore ionomycin for 24 h eryptosis inhibited with PS↓ and Ca^2+^↓				
**Antirhea Borbonica** **Extract (ABE)**	500 mg /L	24–48 h in zebrafish	MGO0–10 mM 24 h	[53]
**Cellular mechanism** At 48 h with ABE eryptosis inhibited with PS↓ and ROS↓. Antiglycative and antioxidant action on albumin with 24 h of treatment				
**Hydroxytyrosol (HT)**	0.1–1 µM [54] 1–5 µM [55]	4 h [54]–24 h [55]	LPA 0.5–2.5 µM—24 h [54] HgCl_2_ 2.5 µM—4 h [55]	[54]
				[55]
**Cellular mechanism** At 24 h with HT 0.1 µM, eryptosis inhibited with PS↓, Ca^2+^↓ and GSH↑. Moreover, FS↑ and ATP↑ with HT 0.5 µM and MV↓ with HT 1 µM [55]. At 4 h with HT 1 µM, eryptosis inhibited with PS↓, GSH↑ and ATP↑ [54]				
**Pyrogallol (PYR)**	2–8 µM	30 min–48 h	Energy depletion—48 h	[56]
			Cell shrinkage—6 h	
			Ionomycin 1 µM—1 h	
			tBOOH 0.3 mM—30 min	
**Cellular mechanism** Eryptosis inhibited with PS↓ with PYR 2 µM at 48 h in presence of energy depletion, at 6 h in presence of hyperosmotic environment, at 1 h in presence of ionophore ionomycin, and also after a treatment for 30 min in presence of oxidative stress inducted by tBOOH				
**Fisetin (FIS)**	15 mg/kg	6 weeks in rats	D-galactose 500 mg/kg 4 months	[57]
		
**Cellular mechanism** Eryptosis inhibited with PS↓, ROS↓, GSH↑				
**Resveratrol (RES**)	1–40 µM	In rabbit 3 h [58]	Cyadox 40 µg/mL—3 h [58]	[58]
		In human 30 min– 48 h [59]	tBOOH 0.3 mM 30 min [59]	[59]
			Energy depletion—48 h [59]	
			Cell shrinkage—48 h [59]	
**Cellular mechanism** In rabbit’s RBCs, at 3 h with RES 40 µM, eryptosis inhibited with oxidative stress↓, activity of active caspases 3 and 8↓ and LDH↓ [58]. Furthermore, in human’s RBCs at 30 min with RES 10 µM, in presence of oxidative stress inducted by tBOOH, PS↓; at 48 h in presence of inducted cell shrinkage with RES 1 µM PS↓ and at 48 h energy depletion PS↓ and Ca^2+^↓ with RES 5 µM and FS↑ with RES 10 µM [59]				
**Naringin (NAR)**	10–40 µM	30 min–48 h	Energy depletion—48 h	[60]
			tBOOH 0.3 mM—30 min	
**Cellular mechanism** At 48 h with NAR, in presence of energy depletion, from 10 µM eryptosis inhibited with PS↓, from 20 µM Ca^2+^↓, and from 40 µM FS↑. At 30 min with NAR, in presence of oxidative stress inducted by tBOOH, eryptosis from 40 µM PS↓ and FS↑				
**Phlorizin (PHL**)	10–100 µM	30 min–48 h	Energy depletion—48 h	[61]
			tBOOH 0.3 mM—30 min	
**Cellular mechanism** At 48 h with PHL 50 µM, in presence of energy depletion, eryptosis inhibited with PS↓ and FS↑. At 30 min with PHL 10 µM, in presence of oxidative stress inducted by tBOOH, eryptosis inhibited with PS↓ and with 50 µM FS↑ and Ca^2+^↓				
**Xanthohumol (XAN**)	0.25–1 µM	30 min–48 h	Energy depletion—48 h	[62]
			tBOOH 0.3 mM—30 min	
**Cellular mechanism** At 48 h with XAN, in presence of energy depletion, eryptosis inhibited with PS↓ with XAN 0.25 µM and Ca^2+^↓ and FS↑ with XAN 0.5 µM. At 30 min, in presence of oxidative stress inducted by tBOOH, eryptosis inhibited with PS↓ with XAN 0.5 µM and Ca^2+^↓ with XAN 1 µM				
**Thymol (THY)**	2.5–20 µg/mL	30 min–48 h	Energy depletion—48 h	[63]
			Cell shrinkage—48 h	
			tBOOH 0.3 mM—30 min	
**Cellular mechanism** At 48 h with THY 20 µg/mL, in presence of energy depletion, eryptosis inhibited with PS↓ and FS↑; with the same conditions but in presence of cell shrinkage THY inhibit eryptosis only with PS↓. At 30 min, in presence of oxidative stress inducted by tBOOH in presence or absence of Ca^2+^ in the ringer, eryptosis inhibited with PS↓ and Ca^2+^↓ with THY 2.5 µg/mL				

**Table 2:** Mechanisms involved in the anti-eryptotic effect of alkaloids compounds against different stimuli in human RBCs.

Phytochemical Compound	Concentration	Treatment Time	Inductor	References
**Caffeine (CAF)**	50–500 µM	48 h	Energy depletion—48 h	[68]
			Cell shrinkage—48 h	
**Cellular mechanism** After 48 h of treatment with CAF 500 µM, in presence of energy depletion, eryptosis inhibited with PS↓, Ca^2+^↓ and FS↑. After 48 h of treatment with CAF 50 µM, in presence of cell shrinkage, eryptosis inhibited only with PS↓				
**Chelerythrine (CHE)**	1–10 µM	24 h	Custonolide 1–80 µM—24 h	[69]
**Cellular mechanism** At 24 h with CHE 10 µM, in presence of custonolide 80 µM, eryptosis inhibited with PS↓ and FS↑				
**Indicaxanthin (IND)**	1–5 µM	48 h	Mixture of oxysterols	[70]
			20 mM—48 h	
**Cellular mechanism** At 48 h with IND 1 µM, in presence of mixture of oxysterols 20 mM, eryptosis inhibited with PS↓, Ca^2+^↓, FS↑, ROS↓ and PGE2↓. Moreover, at 48 h of with IND 2.5 µM GSH↑ and endothelial adherence↓ with IND 5 µM.				

**Table 3:** Mechanisms involved in the anti-eryptotic effect of other natural compounds against different stimuli in human and rabbit RBCs.

Phytochemical Compound	Concentration	Treatment Time	Inductor	References
**Acetylsalycilic acid (ASA)**	50 µM	24 h	4-Hydroxynonenal	[71]
			25–50 µM—24 h	
**Cellular mechanism** Eryptosis inhibited with PS↓ and Ca^2+^↓				
**Tamarind seed**	50–200 µg/mL	24 h	AAPH 100–1000 µM—24 h	[72]
**Extract (TSCEE)**				
**Cellular mechanism** In presence of oxidative stress inducted by AAPH, eryptosis inhibited from TSCEE 50 µg/mL with PS↓, Ca^2+^↓, ROS↓ and GSH↑				
**Plant sterols (MPS)**	22 µM	48 h	tBOOH 0.075 or 0.3 mM 30 min	[73]
			
**Cellular mechanism** Eryptosis inhibited with Ca^2+^↓, Hemolysis↓, ROS↓ and GSH↑ in presence of tBOOH 75 µM.				
**L-Carnitine (LCar)**	200 umol/L	24–48 h	30% uremic serum (*v*/*v*)	[74]
			24–48 h	
**Cellular mechanism** Eryptosis inhibited with PS↓, ROS↓ and GSH↑				
**Salidroside (SAL)**	100–300 µM	24 h	H_2_O_2_ 1 mM—24 h	[75]
**Cellular mechanism** Eryptosis inhibited from SAL 100 µM with PS↓, Ca^2+^↓, ROS↓, eemolysis↓ and activity of active Caspase 3↓				
**Vitamin C (VitC)**	60–280 µM	30 min [77]	H_2_O_2_ 0.5% (*v*/*v*)—30 min [76]	[76]
		30 min–48 h [76]	G6PD deficiency [76]	[77]
			Energy depletion—48 h [77]	
			Cell shrinkage—48 h [77]	
			tBOOH 0.3 mM—30 min [77]	
**Cellular mechanism** In patients with G6PD deficiency, at 30 min with VitC 60 µM in presence or absence of H_2_O_2_ 0.5% (*v*/*v*), eryptosis inhibited with PS↓ and activity of active caspases 3↓ [76]. At 48 h with VitC, in presence of energy depletion, eryptosis inhibited with PS↓, Ca^2+^↓, and FS↑ from VitC 110 µM; with the same treatment but with cell shrinkage, VitC 60 µM inhibit eryptosis with PS↓ and at 30 min, in presence of oxidative stress inducted by tBOOH, eryptosis inhibited with PS↓ [77]				
**Cinnamaldehyde (CIN)**	40 µM	3 h	Cyadox 40 µg/mL—3 h	[58]
**Cellular mechanism** Eryptosis inhibited with oxidative stress↓, activity of active caspases 3 and 8↓ and LDH↓				

## Abbreviations

red blood cells (RBCs); phosphatidylserine (PS); phenolic compound (PC); phospholipase A2 (PLA2); arachidonic acid (AA); prostaglandin endoperoxide synthase (PGHS); cyclooxygenase (COX); Fas receptor (FAS); death-inducing signalling complex (DISC); Fas associated via death domain (FADD); platelets activating factor (PAF); sphingomyelinase (SM); glutathione (GSH); protein kinase C (PKC); end stage renal disease (ESRD); erythropoietin (EPO); chronic kidney disease (CKD); C reactive protein (CRP); systemic lupus erythematosus (SLE); antirhea borbonica extract (ABE); adenosine triphosphate (ATP); fisetin (FIS); forward scatter (FS); hydroxytyrosol (HT); lactate dehydrogenase (LDH); oleoyl-L α-lysophosphatidic acid sodium salt (LPA); methyl glyoxal (MGO); micro vesicles (MV); naringin (NAR); 2- phenethyl isothiocyanate (PEITC); phlorizin (PHL); pyrogallol (PYR); resveratrol (RES); tert-butylhydroperoxide (tBOOH); thymol (THY); wogonin (WGN); xanthohumol (XAN); alkaloids compound (AC); caffeine (CAF); chelerythrine (CHE); indicaxanthin (IND); 2′-azobis(2-amidinopropane) dihydrochloride (AAPH); acetysalycilic acid (ASA); cinnamaldehyde; (CIN); glucose-6-phosphate dehydrogenase; (G6PD); L-carnitine (LCar); mix of plant sterols (MPS); salidroside (SAL); tamarind seed extract (TSCEE); vitamin C (VitC).
